# How to Promote Walking in Women with Fibromyalgia: A Look at Catastrophizing, Goal Conflict, and Avoidance from a Self-Emotional Regulatory Perspective

**DOI:** 10.3390/ejihpe14080142

**Published:** 2024-07-24

**Authors:** Carmen Ecija, Lorena Gutiérrez, Patricia Catalá, Cecilia Peñacoba

**Affiliations:** Department of Psychology, Rey Juan Carlos University, Avda. de Atenas s/n, 28922 Alcorcón, Spain; carmen.ecija@urjc.es (C.E.); lorena.gutierrezh@urjc.es (L.G.); patricia.catala@urjc.es (P.C.)

**Keywords:** fibromyalgia, catastrophism, activity avoidance

## Abstract

The aim of this study was twofold: to explore the concordance between two measures of physical activity (accelerometer and IPAQ) among female fibromyalgia (FM) patients, as well as to examine the impact of psychosocial variables (catastrophism, activity avoidance) on walking among these patients. Helplessness, activity avoidance, and commitment to physical activity were evaluated in 132 FM women. After the first assessment, an accelerometer was placed by a psychologist on each participant for seven consecutive days. Eight days later, accelerometers were collected, and participants were evaluated again using the IPAQ. Two models were tested to analyze mediation and a moderated mediation effect. The mediating role of activity avoidance between helplessness and minutes walked was corroborated regarding the objective measure of walking. The effect of helplessness on minutes walked during a week was mediated by activity avoidance and assessed by accelerometers. Cognitive variables played a contextual role when trying to promote exercise. Activity avoidance must be understood as a key variable in physical activity promotion, specifically in the promotion of walking with the aim to do exercise in individuals with FM.

## 1. Introduction

Fibromyalgia (FM) is a complex multidimensional disorder characterized by chronic diffuse musculoskeletal pain, low physical activity (PA), and a reduced quality of life [1,2,3] that usually leads to changes in the individual’s ability to function physically [4,5,6]. An active lifestyle has been postulated as a resilience factor in FM [7,8], and exercise is one of the more commonly recommended non-pharmacological therapies for this population [2,7,9,10]. Specifically, walking as a type of exercise is easy and accessible, and it has a low musculoskeletal impact [11,12]. Moreover, its effect on pain relief, fatigue, anxiety, depression, number of falls, disability, balance, mobility, and quality of life has been widely demonstrated [11,13,14,15,16].

However, as studies have shown, FM patients rarely meet the guidelines for PA, including walking [17]. According to the fear-avoidance model [18], this could be due to the fact that FM patients frequently avoid walking as a way of minimizing the pain they feel [19]. In this sense, an activity avoidance pattern (AA) is a significant predictor of worse psychological and physical function as well as of greater pain interference in FM patients [20]. However, pain is not the only factor that explains low adherence to walking. The previous literature has shown that although AA implies fewer minutes spent walking, other factors also come into play, such as the conflict between PA and patients’ perceptions of it, influencing well-being and quality of life in individuals with FM [21]. This has been observed specifically in the case of walking as a form of physical exercise [21,22,23].

Within the fear-avoidance model [18] regarding the chronification of pain, the motivational perspectives of goal conflict and catastrophizing play a key role. Catastrophizing has been proposed as one of the most important psychosocial predictors of pain, distress, and disability in individuals with FM [24,25,26,27,28,29]; it has a specific predictive power within the interpersonal context in which pain occurs [30]. In general terms, it entails processing pain through heightened (rumination) or exaggerated thoughts (magnification), which involve fixating excessively on the painful sensation, amplifying its severity and perceiving it as beyond control (helplessness), making it a pivotal factor influencing symptom severity. From a motivational perspective, catastrophizing could be defined as the result of the interference between attaining pain-controlling goals and other relevant goals [31,32]. Patients with chronic pain usually anticipate negative consequences from physical activity (as a possible relevant goal), which leads to avoiding activities that could prevent pain [33]. Along with this, the preference for completing goals has also emerged as a relevant factor in undertaking and maintaining function in patients with FM and has become a recent focus of research from contextual models [34] in which goals have been underscored as a way to explain different profiles of women with FM [35,36,37,38,39,40].

On the one hand, taking into consideration that psychological factors influence the relationship between chronic pain and disability—with behavioral (activity avoidance) and cognitive (catastrophizing and goals) factors being the most explored [41]—and, on the other hand, the relevant role of the clash between how people with chronic pain feel about their abilities and how they behave [42], there is a need to delve into the assessment and implementation of multicomponent interventions including both objective and subjective measures, specifically those related to maintaining patients’ functioning despite ongoing pain [43]. Walking cannot be reduced solely to the patient’s capacity or to mere observable motor behavior; it is necessary to analyze this behavior in the context in which it takes place, considering the effect of cognitive variables such as catastrophizing (and specifically hopelessness) and goal preference on PA while taking into account the influence of activity avoidance [27,38,44,45,46,47].

Biopsychosocial models posit that how individuals think and behave in relation to their pain plays a critical role in their pain experience, highlighting people’s abilities to effectively (or not) manage pain. In this context, hopelessness and activity avoidance have been classified as inherently maladaptive variables related to escaping from activities that are anticipated to be unpleasant or pain-eliciting [48], establishing activity avoidance as a highly relevant predictor of disability [49]. This is in agreement with the framework of the behavioral activation (BA) approach, which suggests that if the individual’s environment provides contact with reinforcement for unhealthy behaviors and lacks reinforcement for healthy behaviors, unhealthy behaviors will increase and healthy behaviors will decrease [50]. Based on this, we can hypothesize that catastrophizing, and specifically helplessness, may influence FM through the contextual role of activity avoidance. This could be in accordance with contextual–functional approaches [34] that put an adapted behavior to the service of a set personal goal [51]. Within this more integrative perspective, the effects of helplessness and avoidance on physical activity, specifically on walking, could be related to underlying personal goals and other contextual factors that need to be explored [52].

Therefore, the current study had two main objectives. The first was to analyze the concurrence between two measures of physical activity (accelerometer and IPAQ), focusing on the tendency to avoid walking among female FM patients. The second was to explore the role of the psychosocial variables discussed above (helplessness and activity avoidance), taking into account the contextual role of the tendency to avoid walking when pain appears. Meanwhile, walking was measured from the perspective of the emerging goal conflict (both from the patient’s perception and actual behavior). We believe that the results from this study may be useful in healthcare contexts related to the implementation of effective treatments of physical activity in patients with FM, including both measures of actual performance and patient perception.

## 2. Materials and Methods

### 2.1. Participants

A sample of 132 women with FM were recruited from different FM mutual aid associations in the Communities of Madrid and Castilla-La Mancha (Spain), all having been diagnosed according to the American College of Rheumatology (ACR) criteria [1,53,54]. The occurrence of FM shows a variation based on gender, with it being more common in females. However, the prevalence in males can vary depending on the study methods used. A 2017 study by Marques and others [27] indicated that FM’s prevalence in the general population across various countries spanned between 0.2% and 8.8%, with the rates for women lying between 2.4% and 6.8%. In accordance with these percentages, only women were included in our study, while the following additional criteria were included: being above 18 years old, providing written consent to participate, having a medical prescription to walk, and not presenting any physical impairments to carry out physical activity.

### 2.2. Procedure

The University Committee on Ethics approved this study. Based on the prospective nature of our study, two different assessments were conducted, with a one-week separation between each. The participants first gave their informed consent to take part in the project and were then given a booklet of questionnaires (this booklet contained questions from the measures noted below), which took 20–30 min to complete in their associations in the presence of a psychologist. Firstly, participants self-reported their sociodemographic and clinical data using an ad hoc questionnaire (pain). Then, using validated questionnaires, participants were evaluated regarding helplessness (pain catastrophizing), activity avoidance (AA), and commitment to physical activity (PA). Finally, after finishing the first assessment, levels of physical activity were evaluated and an accelerometer was placed by a psychologist on the wrist of each participant for 7 consecutive days, starting on the same day that they received the accelerometer. Participants were instructed to always wear the accelerometer, except during water-based activities such as showering or swimming. After 8 days, the accelerometers were collected in a second evaluation time, and participants were given a second booklet that included the IPAQ to be answered individually by each participant. The data from the accelerometers was finally downloaded onto a computer and processed using the manufacturer software.

### 2.3. Measures

#### 2.3.1. First Assessment

Helplessness (H). The Spanish adaptation of the Pain Catastrophizing Scale (PCS) evaluates three components of pain catastrophizing. The Pain Catastrophizing Scale (PCS) stands out as the most widely utilized tool for assessing pain catastrophizing [30]. Its development was based on a multidimensional understanding of catastrophizing, encompassing aspects of magnification (e.g., pondering whether something serious might happen), rumination (e.g., unable to stop thinking about it), and helplessness (e.g., feeling powerless to alleviate pain intensity). It contains 13 items in a 4-point Likert response format [55,56]. In this study, the helplessness subscale was used. Specifically, we measured when women were feeling powerless to alleviate pain intensity. This subscale contains 6 responses, such as “there is nothing I can do to reduce the intensity of the pain”. Higher scores on this scale represent higher helplessness. In the present study, the internal consistency of the helplessness subscale was 0.89.

Activity avoidance (AA). This activity pattern was measured through the Spanish version of the Activity Patterns Scale (POAM-P) [57]. The POAM-P (24 items on a 5-point Likert scale divided into 8 dimensions) allows assessment of three widely accepted general activity patterns (avoidance, persistence, and pacing). Avoidance patterns include pain avoidance (avoidance of a behavior in the presence or anticipation of changes in pain) and activity avoidance (avoidance refers to the patients’ condition of being in pain rather than the fluctuating pain experience). In this study, the activity avoidance subscale was used. Studies so far support the validity of the Activity Patterns Scale to measure different activity patterns in chronic pain settings [57,58]. In the present study, the Cronbach alpha for the activity avoidance subscale was 0.82.

Avoidance goal (AG). Walking with the goal of avoiding physical exercise. The preference for pain-avoidance goals in relation to activities was assessed using the Spanish adaptation [39] of the Goal Pursuit Questionnaire for Pain (GPQ-P) [59]. The GPQ measures the goal pursuit of people with pain, considering achievement goals (performing activity) and hedonic goals (avoiding activities because of pain), which can be activated simultaneously or be in conflict. The adaptation of the GPQ to physical exercise was used specifically in relation to 5 specific situations: (1) walking while doing other activities, (2) walking with the goal of physical exercise, (3) mild physical activity, (4) moderate physical activity, and (5) intense physical activity. Higher mean scores in each factor indicate stronger preferences for a hedonic goal in comparison to an achievement goal, that is, to avoid pain (Factor I) or to maintain a positive mood (Factor II). For this study, the “Pain avoidance goals” factor was selected. Cronbach’s alpha in this study was 0.81.

Pain severity (covariate). Pain severity was assessed by the mean score of the four pain severity items (maximum, minimum, overall pain intensity during the last 7 days, and pain intensity at the current time) from the Brief Pain Inventory (BPI) [60]. Each rating was evaluated using an 11-point numerical scale from 0 = “no pain” to 10 = “the worst pain you can imagine”. This procedure has been widely used in the pain-focused literature [61]. Researchers have shown an excellent internal consistency in previous studies of the Spanish population with chronic pain (α = 0.93) [62]. In this study, the internal consistency of this scale was 0.86.

#### 2.3.2. Second Assessment: Physical Activity Measures

Regular walking behavior (IPAQ-walking). During the second evaluation, the Spanish version of the International Physical Activity Questionnaire (IPAQ) was used to assess the physical activity levels of patients. This questionnaire catalogs various activities undertaken in the past week, noting their intensity, duration, and frequency. From this, each participant’s physical activity level was determined based on the IPAQ’s data processing and analysis guidelines. High activity levels were defined as at least three days of vigorous activity totaling 1500 or more MET (metabolic equivalent of task) minutes weekly, or seven days of any combination of walking, moderate, or vigorous activities totaling at least 3000 MET minutes. Moderate activity levels were achieved by engaging in vigorous activities and/or walking for at least 30 min per day for three days, or moderate activities and/or walking for the same duration over five days, accumulating 600 or more MET minutes. Participants who did not meet the criteria for high or moderate levels were considered to have low activity levels. The IPAQ version used in this study was validated within the Spanish population, showing strong reliability in measuring total physical activity, vigorous and moderate activities, and walking time [63].The internal consistency for our sample was also robust, with Cronbach’s alpha values reflecting the reliability of the vigorous, moderate, and walking activity measures (0.871 in vigorous activity, 0.721 in moderate activity, and 0.753 in walking activity). For the present study, MET-mins related to walking behavior were used. Additionally, to analyze sedentary behavior, the questionnaire included questions about the amount of time spent sitting down on a working day.

Physical activity (St; steps). ActiGraph wGT3X-BT (Pensacola, FL, USA) was used to quantify physical activity objectively by measuring accelerations in physical activity from three dimensions (anteroposterior, mediolateral, and longitudinal axis) (CE., 2005). It is measured in counts per minute, which is then translated to METs. Measurements can range from 1 to 18 METs, depending on the intensity of physical activity [22]. A total of 7 continuous days with a minimum of 10 valid hours per day was required for being included in the study analysis. Participants were instructed to wear an Actical accelerometer on their hip for up to 8 days, and to always wear it, except when performing water-based activities. Episodes of 20 continuous minutes with intensity 0 were excluded from the analysis. The Actical accelerometer has been validated to measure physical activity in adults (DP, 2006). We calculated mean counts per minute by dividing the sum of total counts per epoch for a valid day by the number of minutes of wear time on that day across all valid days. Time spent walking was consequently defined as minutes accumulated between 100 and 759 counts min^−1^. We used the manufacturer software (ActilifeTM v.6.11.7 desktop) for data download, reduction, cleaning, and analysis purposes. Further details are available elsewhere [64]. The average minutes per day as a walking variable was used in the analyses.

### 2.4. Data Analysis

**Figure 1:**
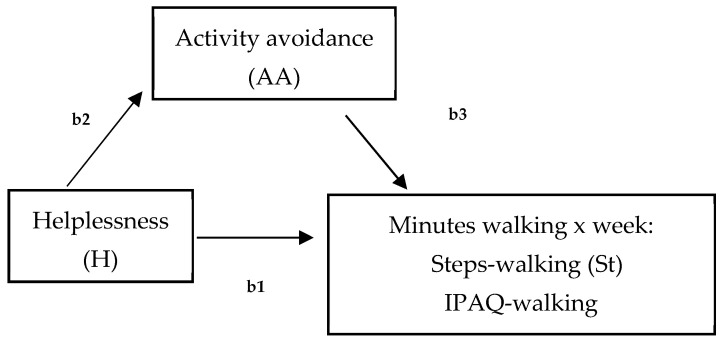
Hypothesized model of the mediated effect of helplessness on minutes walking (per week), measured by steps-walking (St, accelerometer) and IPAQ-walking by activity avoidance (b1 = total effect; b2 = direct effect; b3 = indirect effect).

**Figure 2:**
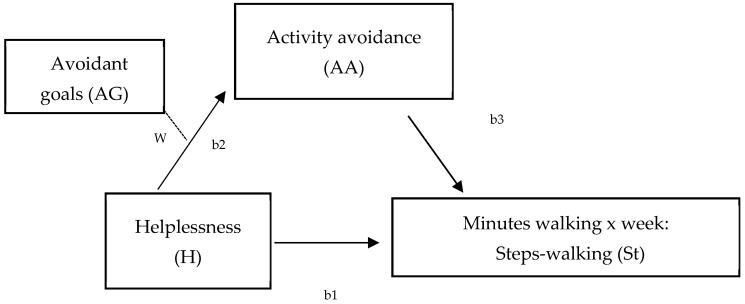
Hypothesized model of the moderated mediation effect of hopelessness on minutes walking (per week), measured by steps-walking (St, accelerometer) at different levels of avoidant goals (W) (W = moderation effect; b1 = total effect; b2 = direct effect; b3 = indirect effect).

Data were analyzed using SPSS 27 (Windows). The characteristics of the sample were described using frequency distributions, percentages, and mean and standard deviations. Following the first aim of this study, differences between walking-IPAQ and accelerometers scores were assessed using the Bland and Altman method [65]. Second, bivariate Pearson correlation analyses were performed to assess the relationships between both measures of physical activity (walking) and hopelessness, avoidance goals regarding walking, activity avoidance, and pain. Statistical significance was established at a *p* value of less than 0.05. Third, two models were tested by the SPSS macro-PROCESS macro 3.5 to analyze mediation (Figure 1), and a moderated mediation model (Figure 2) (model 7) between variables. First, two mediation analyses were run to examine the mediation role of activity avoidance among hopelessness and both physical activity measures (accelerometer and IPAQ) (model 4, Figure 1). To test these two models, regression (to calculate statistics for specific paths) and bootstrapping (to generate a confidence interval [CI] for the mediation effects) analyses were used. Variables were centered before the analyses by subtracting the mean from the predictors to rescale them. It revealed the effect when the remaining predictors had a value of ‘0’ [66] and created ‘artificial’ scores by rescaling the predictors. However, centering had no effect on model fit, significance tests, and standardized slope values. Trying to analyze effects described altogether, a moderated mediation model (model 7, Figure 2) was run. This model tested if the indirect effect of helplessness on physical activity (accelerometer), by way of activity avoidance, depended on avoidance goals regarding walking. Product terms of helplessness (centered) × avoidance goals regarding walking (centered) were added to the regression models predicting physical activity. Simple slope analyses were performed to illustrate significant interaction effects [67]. The index of moderated mediation was estimated as a measure of the association between an indirect effect and a moderator, together with a 95% confidence interval, from bootstrapping 10,000 samples [68]. Pain was specifically considered as a significant covariate in each model, controlling also for sociodemographic covariates that were related to the variables of interest.

## 3. Results

### 3.1. Participant Characteristics

The sample had a mean age of 56.91 years (SD = 8.9) (ranging between 30 and 78 years old). Related to employment status, 12% were employed (all of them part-time; mean of working hours = 5.49; SD = 2.92), 33.8% were homemakers, 32% were retired (18.8% reported chronic pain as a reason for retirement), 12.1% were unemployed, and 10% were employed but on sick leave. Half of the women were married (53%) or in a stable relationship. Regarding education level, 52.6% of women had completed elementary school as their highest level of education (according to the International Standard Classification of Education (ISCED, 2011). The average time since their fibromyalgia diagnosis was 12.14 years (SD = 8.45), ranging between 1 and 46 years. Finally, the pain mean BPI score was 7.15 (SD = 1.52).

### 3.2. Descriptive Data and Correlations

Table 1 shows Pearson correlations between the variables included in the analysis. Hopelessness was positively correlated with activity avoidance (r = 0.25; *p* = 0.007), and activity avoidance was negatively correlated with steps assessed by accelerometer (r = −0.32; *p* = 0.02). Regarding pain, positive correlations were found with hopelessness (r = 0.30; *p* = 0.001) and negative correlations with activity avoidance (r = −0.32; *p* = 0.02). No significant correlations were found related to exercise goals and regular walking with hopelessness, steps, activity avoidance, and pain.

### 3.3. Differences between Physical Activity Measures: Accelerometer and Regular Walking

Following the first aim of this study, the results from differences estimated by the Bland and Altman method show significant variations in measurements between the IPAQ and the accelerometer in all physical activity intensities, including walking behavior [65]. Specifically related to their regular walking behavior (Table 2), steps measured by the accelerometer show higher minutes registered compared to the IPAQ. Moreover, although correlations with physical exercise intensities were significant, the most significant correlation was related to walking intensity (*p* = 0.008).

### 3.4. Test of the Models

First, the mediating role of activity avoidance (AA) between helplessness (H) and physical activity measured by steps (St) (accelerometer) and IPAQ-walking was examined with pain as the covariate (Figure 1). For both mediating models, no significant total effects of the predictor (hopelessness) on steps (B = −7.95, SE = 5.55, t = −1.43, 95% CI = [−19.16; 3.21], *p* = 0.15) or on IPAQ-walking (B = 5.74, SE = 17.93, t = 0.04, 95% CI = [−28.44; 42.96], *p* = 0.68) were observed. However, focusing on the mediating effect of activity avoidance, the effect of hopelessness on steps was totally mediated by activity avoidance (B = −5.75, SE = 3.47; 95% CI = [−13.58, −0.05]), while activity avoidance did not have a mediated effect when IPAQ-walking was evaluated as a dependent variable (B = −1.51, SE = 4.90, 95% CI = [−12.21, 8.01]). That is, hopelessness predicted higher activity avoidance, which in turn predicted fewer minutes walked when evaluated by an accelerometer in our sample of women with FM (Figure 1).

### 3.5. Moderated Mediation Model of Openness to Experience on Activity Acceptance by Positive Effect at Different Levels of Mood Management Goals

Second, a moderated mediation model was conducted to test the indirect effect of hopelessness on physical activity (measured by accelerometer) by activity avoidance at levels of avoidance goals regarding walking and pain as covariates (Figure 2). The results show that the effect of hopelessness on activity avoidance varied at different values of avoidance goals regarding walking (Hayes’ algorithm, estimated for physical activity, was ß = −1.67 [95% CI = −4.48/−0.003]), an effect that was maintained when physical activity measured by accelerometer was included as a dependent variable in the model. Particularly, helplessness mediated by activity avoidance was significantly associated with physical activity when levels of avoidance goals regarding walking were medium (Value: 0.48; ß = −5.77, [95% CI = −14.37/−0.01]) and high (Value: 2.48; ß = −9.12, *p* < 0.001, [95% CI = −22.01/−0.36]). This result (Table 3, Figure 2) shows that the strength of the relationship between hopelessness and physical activity (measured by minutes of walking per week by the accelerometer) via activity avoidance increased at higher levels of avoidant goals. Thus, the indirect effect of helplessness on minutes of walking measured by accelerometer via activity avoidance was higher in individuals who placed a higher importance on exercise.

## 4. Discussion

The present study had two specific objectives. First, based on differences found by researchers between measures related to walking behavior in women with FM [49,69,70], our first objective focused on evaluating the concurrence between two measures related to physical activity—one objective (accelerometer) and one subjective (IPAQ), both selected to evaluate the minutes walked for one week (Table 1). These results are partially in agreement with studies that have shown that physical activity perception in patients with FM is lower compared to activity performed when assessed by objective measures [69,70]. In accordance with recent studies, our results show that, in relation to low-intensity physical activity (walking), both measures show the highest correlation compared to the assessment of sedentary, moderate, or intense activity, assessed using the same measures [22]. It appears that, although our results corroborate this effect for sedentary, moderate, and intense measures of physical activity, the same is not true for walking. Women with FM walk more minutes during a week than they perceive themselves to walk. As previous studies have shown, the perceived physical function may be even worse than the objective physical function among women with fibromyalgia [10]; thus, the impact on the self-confidence about what they are and are not able to do is largely affected, which in turn is related to the concept of self-efficacy. Our objective measurement results may be linked to the fact that women engage in walking behavior as part of their daily routine. However, since this is not undertaken with the specific intention to perform physical exercise, and because they perceive they are not able to exercise (low self-efficacy about physical activity), this activity is not recognized as such [58].

Based on our results and trying to determine how objective and subjective associations differ [70], our second aim focused on the analysis of the differential role of psychological and contextual variables on perception and actual performance. In line with this aim, despite the fact that helplessness, goal conflict, and activity avoidance have been identified as contextual factors to be taken into account in the assessment of physical activity in women with FM, there are no studies to date analyzing these variables together in relation to walking, or aiming to analyze differences when assessing behavior using different measures (objective and subjective). Therefore, following this aim, two models were analyzed. Firstly, one model was focused on analyzing if activity avoidance mediated the relationship between hopelessness and minutes walked during a week, assessed by both measures (Figure 1). Secondly, we analyzed if the mediated effect of activity avoidance between hopelessness and walking was influenced by levels of avoidant goals. The results show interesting data. On the one hand, the mediating role of activity avoidance between hopelessness and minutes walked was corroborated, but only regarding the objective measure of walking behavior assessment. This is in agreement with a recent study where, compared to self-report measures, only step count significantly changed across time, with mean steps peaking at eight weeks [71]. It appears that the effect of helplessness on minutes walked during a week occurs when totally mediated by activity avoidance, and, specifically, when minutes walked are assessed using an accelerometer. Moreover, the fact that higher levels of hopelessness imply greater activity avoidance, which in turn negatively influences minutes walked, is in accordance with a recent study that focused on cognitive variables characterizing FM patients who found pain catastrophizing and, specifically, hopelessness to be a significant predictor of adaptability in a Spanish FM sample [72]. This result shows that pain avoidance is especially dysfunctional in patients with high helplessness. Catastrophizing thoughts about pain are characterized as an exaggerated negative mental stance linked to the real or anticipated experience of pain [30]. As explained above, this notion is empirically structured into three dimensions: magnification, rumination, and helplessness. Magnification is the excessive perception of pain situations and pessimistic expectations regarding the progression of pain, while rumination encompasses repetitive thoughts, worry, and the inability to suppress such thoughts. Finally, helplessness (our focus of study) is the perceived lack of control over these thoughts [56]. Most studies challenge the uniformity of this concept, suggesting that each dimension has distinct effects [24,28]. For instance, it has been noted that shifts in helplessness correlate with improvements across all disease variables, whereas alterations in rumination and magnification have varied impacts on different disease outcomes. On the one hand, these results show that cognitive variables have a basic contextual role when trying to promote exercise, a role that must necessarily consider activity avoidance as a key variable in the promotion of physical activity and, specifically, the promotion of walking with the aim to do exercise in women with FM. This is congruent with studies of the model of fear of movement that have shown that activity avoidance is the highest predictor of FM impact [49,73] and, specifically, that a low level of physical activity is influenced by self-efficacy, mainly through activity avoidance [39]. On the other hand, within the framework of the behavioral activation (BA), if the individual’s environment provides contact with reinforcement for unhealthy behavior and lacks reinforcement for healthy behavior, unhealthy behavior will increase and healthy behavior will decrease [50]. Thus, in a clinical context, the prevention of activity avoidance may be a key factor in walking when it deprives women with FM of the opportunity to obtain reinforcement through the walking behavior itself, which in turn may be maintaining the avoidance of activity by promoting the negative perception that women have about their physical activity [74,75,76]. We can conclude that promoting walking is necessary to improve levels of hopelessness, preventing women from entering an activity avoidance pattern. Women need the opportunity to obtain reinforcement from their own behavior, improving their self-efficacy with regard to their functional capacity.

Secondly, our results show that helplessness plays a significant role in minutes spent walking via the mediated effect of activity avoidance. This result is in concurrence with a recent study in which catastrophizing had a residual importance on physical activity also mediated by a contextual variable, such as psychological flexibility [77]. As Vallejo et al. (2021) have pointed out, contextual variables should be considered for evaluation and early intervention in FM patients. Patients are faced with multiple competing goals, and they should prioritize between those related to avoiding pain and those related to maintaining or incorporating other goals different from pain control [78,79,80,81]. Our results show that in women with FM, hopelessness could be another cognitive variable that influences walking via the mediated role of activity avoidance.

This study presents several limitations. Initially, its cross-sectional nature hinders the determination of causality [22]. Additionally, the research focuses solely on women with fibromyalgia (FM), necessitating further studies among males and other pain-affected demographics to confirm the applicability of the results. Although fibromyalgia tends to affect females more frequently (often surpassing 90% prevalence), the correlation between fibromyalgia and gender remains contentious and intricate due to various factors. These factors encompass variances in diagnostic criteria and methodologies, which often results in the underdiagnosis of male patients with fibromyalgia and the overdiagnosis of females in both clinical and research settings [1,2]. Additionally, studies into disparities in symptoms and psychological variables have yielded inconclusive results [5], underscoring the necessity for future studies to separately assess females and males afflicted with fibromyalgia. Lastly, the recruiting method for the participants through mutual-aid pain associations might also skew the sample toward women, potentially not representing the usual cases in secondary care. It is important to note, however, that these types of associations are prevalent among Spanish FM sufferers, and similar clinical and sociodemographic profiles have been observed across various settings [39]. Despite exploring numerous clinical and psychosocial factors, this study does not cover all possible aspects relevant to FM [3].

Nevertheless, considering these factors and recent research indicating that physical activity (PA) affects individual variances in FM symptoms and overall functionality, this study contributes to the evidence supporting interventions that enhance openness to experiences, positive effects, and mood management. These interventions aim to foster pain acceptance, a crucial element in chronic pain management that could significantly improve patient quality of life [82].

In terms of practical application, our findings support a burgeoning field of intervention that utilizes positive psychology in chronic pain populations. This approach seeks to amplify positive emotions, thoughts, and actions [83]. Positive psychology interventions (PPIs) adopt a resource-oriented strategy that emphasizes fortifying the positive traits of individuals, which could be advantageous in the management of chronic pain.

Our findings provide healthcare practitioners with fresh psychological markers that can enhance collaborative care approaches for affected individuals. Considering that there are modern comprehensive pain management programs emphasizing the development of adaptive coping strategies, this study offers a new perspective for healthcare workers, particularly nurses, to tailor their care strategies in fibromyalgia (FM) toward embracing pain, fostering positive emotions, and managing one’s mood instead of concentrating solely on diminishing pain sensations. However, further research is essential to definitively determine the cause-and-effect links among these factors.

## 5. Conclusions

From a clinical point of view, health professionals should consider cognitive and behavioral patterns as psychological variables when encouraging walking behavior. It has been demonstrated that hopelessness does play a role in walking via activity avoidance, specifically in women who consider exercise an important behavior for battling their disease. These results yield significant data within the panorama of FM treatments and, of course, in relation to the adherence, promotion, and prevention of the disease. It appears that helplessness and activity avoidance influence minutes spent walking, as assessed through an objective measure in women who place a high importance on exercise. It seems to be that helplessness has a distinct impact on the behavior of walking as assessed by an objective measure. This influence manifests through the avoidance of activity, particularly in individuals who possess higher levels of hedonic goals, which involves shunning activities due to pain. It can be concluded that promoting exercise among women with fibromyalgia (FM) is crucial, considering not only the amount of exercise but also how it is performed. Helplessness and activity avoidance are measured against the goals women set for their activity levels. Therefore, promoting the work on contextual variables as a preventive measure, and for identifying profiles in women with FM, becomes a successful goal. This is because these variables directly and indirectly influence the ability to exercise and, consequently, the well-being of women with FM.

## Figures and Tables

**Table 1:** Pearson correlations between study variables.

	2	3	4	5	6
**1.** Helplessness	0.25 **	−0.16	−0.01	−0.07	0.30 **
**2.** Activity avoidance		−0.32 *	−0.05	−0.01	−0.32 *
**3.** Minutes walked (week) by steps			0.11	0.08	0.21
**4.** Minutes walked (week) by regular walking				0.002	−0.61
**5.** Avoidance goal					0.15
**6.** Pain					1

* *p* < 0.05; ** *p* < 0.01.

**Table 2:** Descriptive physical activity according to intensity of IPAQ and ActiGraph wGT3X-BT in a week.

	Sedentary Behavior		Walking		Moderate		Vigorous	
	IPAQ MET min Day^−1^	Accelerometer Counts·min^−1^	IPAQ MET min·Day^−1^	Accelerometer Counts·min^−1^	IPAQ MET min·Day^−1^	Accelerometer Counts·min^−1^	IPAQ MET min·Day^−1^	Accelerometer Counts·min^−1^
	496	18,567	636	1019	225	547	63	22
*p*	0.017		0.008		0.027		0.047	

**Table 3:** Moderate mediation model: regression of hopelessness, activity avoidance (mediator) on minutes walked measured by steps (St), and avoidance goals regarding walking (moderator).

	B (SE)	t	*p*	[LLCI-ULCI]
VI: Helplessness (H)	0.18 (0.06)	2.97	0.004	[0.05/0.30]
M: Avoidance goals (AG)	0.24 (0.15)	1.61	0.11	[−0.62/0.02]
H × AG (interaction)	0.06 (0.02)	2.51	0.01	[0.01/0.11]
* Pain (covariate)	0.13 (0.19)	−0.67	0.50	[−0.40/0.55]
Conditional effects of the focal predictor (H) at values * of the moderator (AG) on positive effect				
−2.51	−0.02 (0.08)	0.30	0.76	[−0.15/0.20]
0.48 *	0.21 (0.06)	3.44	0.001	[0.08/0.33]
2.48 *	0.33 (0.08)	3.88	0.0003	[0.16/0.50]
Regression of helplessness and activity avoidance (AA) on steps (St)				
	B (SE)	t	*p*	[LLCI-ULCI]
Helplessness (H)	−2.21 (6.13)	−0.36	0.71	[−14.59/10.15]
Activity avoidance (AA)	−26.9 (13.81)	−1.95	0.05	[−54.81/0.88]
* Pain (covariate)	−0.30 (18.12)	−0.01	0.98	[−36.85/36.23]
Model summary	R^2^: 0.12			
Indirect effects at values * of AG				
−2.51	−0.74 (3.08)			[−6.82/6.07]
0.48 *	−5.77 (3.74)			[−14.37/−0.01]
2.48 *	−9.12 (5.53)			[−22.01/−0.36]
Indexes of moderated mediation	−1.67 (1.16)			[−4.48/−0.003]
Pairwise contrast between conditional indirect effects (Effect 1 minus Effect2)				
	Effect 1	Effect2	contrast	[LLCI/ULCI]
	−5.77 −9.12 −9.12	−0.74 −0.74 −5.77	−5.03 −8.38 −3.35	[−13.46/−0.009] [−22.44/−0.01] [−9.97/−0.006]

Notes: Models include controls for age, sex, education level, employment status; conditional effects of the focal predictor at values * of the moderator (AG); indirect effects of helplessness (H) on activity avoidance (AA) at values * of AG. Abbreviations: BootLLCI, bootstrapping lower limit confidence interval; BootULCI, bootstrapping upper limit confidence interval; SE, standard error. Model 7 from Process.

## Data Availability

The data presented in this study are available upon request from the corresponding author.
